# Giant duplication of the transverse colon in an adult: A case report and literature review

**DOI:** 10.1515/biol-2022-0626

**Published:** 2023-06-14

**Authors:** Zhihao Peng, Mingkai Zhang, Ruixue Wang, Hai Huang, Zongke Sun, Yanbin Li

**Affiliations:** Department of Emergency Surgery, Binzhou People’s Hospital, Binzhou 256601, Shandong, China; Department of Gastrointestinal Surgery, Binzhou Medical University Hospital, Binzhou 256603, Shandong, China; Department of Pediatric Hematology and Endocrinology, Binzhou Medical University Hospital, Binzhou 256603, Shandong, China; Department of Burns and Plastic Surgery, Binzhou Medical University Hospital, Binzhou 256603, Shandong, China

**Keywords:** transverse colonic duplication, adult, ileus, emergency surgery, previous laparotomy

## Abstract

Intestinal duplication is a rare congenital malformation that can occur in any segment of the digestive tract. It is most commonly found in the ileum of infants and is rarely reported in adults, especially in the colon. Diagnosing intestinal duplication can be extremely challenging due to its diverse clinical manifestations and complex anatomical structure. Surgical intervention is currently considered the mainstay of treatment. In this report, we presented a case of giant duplication of the transverse colon in an adult.

## 1 Introduction

Intestinal duplication is a rare congenital malformation. The segments of repeated bowel are firmly attached to or share a common wall with the gastrointestinal tract and might have a common blood supply with the adjacent segment of the bowel. More than 80% of the cases are found due to acute abdominal and intestinal obstruction symptoms before the age of 2 years, and diagnosis in adulthood is rare [1]. Intestinal duplication can occur in any segment of the digestive tract, with the small intestine, particularly the ileum, being the most common site [2,3,4].

Intestinal duplication encompasses two main histological types: cystic duplications, which do not communicate with adjacent bowel segments and account for more than 80% of the cases, and tubular duplications, which are less common and have communication with adjacent bowel segments [5]. The clinical manifestations of intestinal duplication are diverse and can include symptoms such as vomiting, abdominal distension, abdominal mass, and black stool. However, these symptoms are non-specific and can also be seen in other gastrointestinal conditions. Due to the complex anatomical structure, diagnosing this condition is extremely challenging [1,3,6]. The expertise and knowledge of competent doctors are crucial in identifying these diseases. Currently, surgical resection is considered the primary treatment for the digestive tract duplication. In this study, we described the clinicopathological features of a rare case involving a giant transverse colonic duplication in an adult patient. Moreover, we also discussed the diagnosis, differential diagnosis, and treatment of intestinal duplication in detail, in conjunction with relevant literature.

## 2 Case report

### 2.1 Patient information

A 52-year-old woman presented to our department with persistent upper abdominal pain that had already lasted for 20 h. She also reported experiencing nausea, vomiting, and cessation of flatus and defecation. However, there were no significant findings of radiation pain, high fever, black stool, or other suggestive symptoms. The patient had a medical history of diabetes for 3 years and underwent partial small intestine resection 22 years ago due to a traffic accident. She denied any allergies or a history of illicit drug or alcohol use.

During the physical examination, the patient’s abdomen was noticeably swollen, and tenderness was observed in the upper abdomen and umbilical region. Laboratory tests revealed elevated white blood cell count (15.0 × 10^9^/L), neutrophil percentage (91.1%), and glucose level (9.66 mmol/L), indicating potential infection and hyperglycemia. However, tumor markers and other hematological features were normal.


**Informed consent:** Informed consent has been obtained from all individuals included in this study.
**Ethical approval:** The research related to human use has been complied with all the relevant national regulations, institutional policies, and in accordance with the tenets of the Helsinki Declaration and has been approved by Binzhou Medical University Hospital Clinical Trial Ethics Committee.

### 2.2 Imaging findings

**Figure 1:**
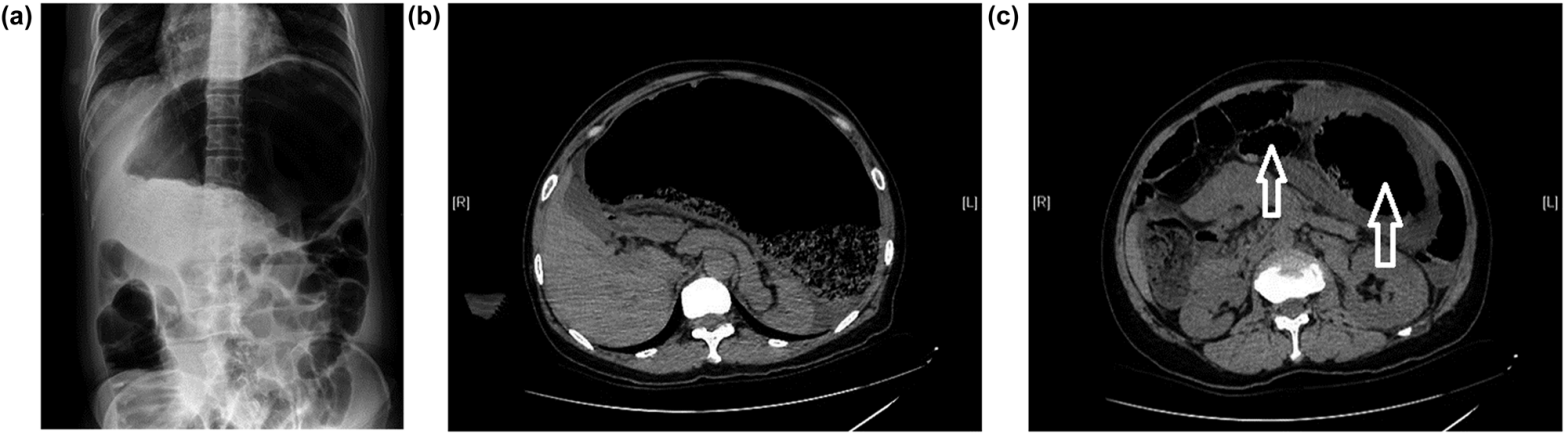
Imaging examination of the transverse colonic duplication. (a) Abdomen film with the patient in the erect position. (b) CT scan shows a giant cyst in the abdominal cavity. (c) The arrows indicate the transverse colon and duplication.

The computed tomography (CT) scan revealed a large, uniform, low-density shadow with a dilated intestinal tube and multiple air-fluid levels. The maximum diameter of the expansion was approximately 25.8 cm, with partially observed sieve-like high-density filling. The intestinal wall was significantly thickened, and the border with the nearby colon was blurred and difficult to distinguish. Adjacent abdominal organs, such as the stomach, spleen, liver, and pancreas, were displaced by compression (Figure 1). Due to the patient’s history of previous surgery and symptoms of intestinal obstruction, an endoscopy could not be performed, making it challenging to establish an accurate preoperative diagnosis. Initially, we considered the possible diagnoses of a colonic diverticulum, megacolon, and congenital intestinal malformation.

### 2.3 Therapeutic interventions

**Figure 2:**
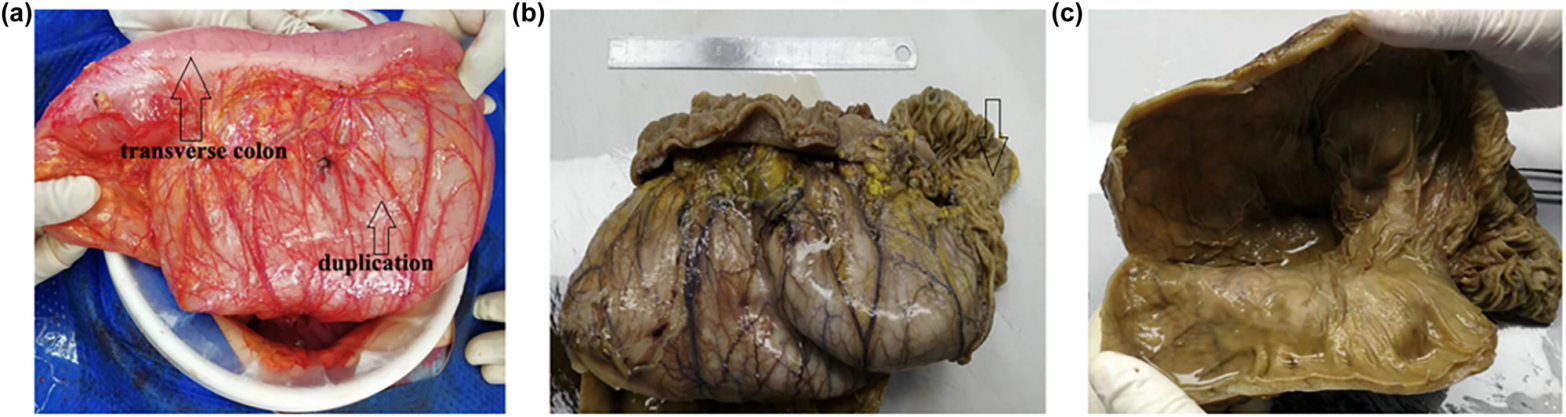
Postoperative specimen. (a) Surgical exposure of the transverse colonic duplication. (b) Removal of the transverse colonic duplication; the marked points in the figure are the locations where intestinal canals communicate. (c) Multiple cut surfaces of the transverse colonic duplication.

Given the severity of the patient’s condition and the history of intestinal obstruction and abdominal surgery, an exploratory laparotomy was deemed necessary. During the surgery, we observed obvious dilation of the small intestine, with a cystic mass on the lateral mesentery. The structure appeared bag-like on the surface, with an unclear boundary with the transverse colon wall and obvious expansion. Due to the excessive intestine expansion, pulling it out from the incision was difficult. As a result, we decompressed the cyst by creating a small breach, disconnected the transverse colon at a distance of 10 cm from the cyst, and performed a side-to-side anastomosis (Figure 2).

### 2.4 Histopathological findings

**Figure 3:**
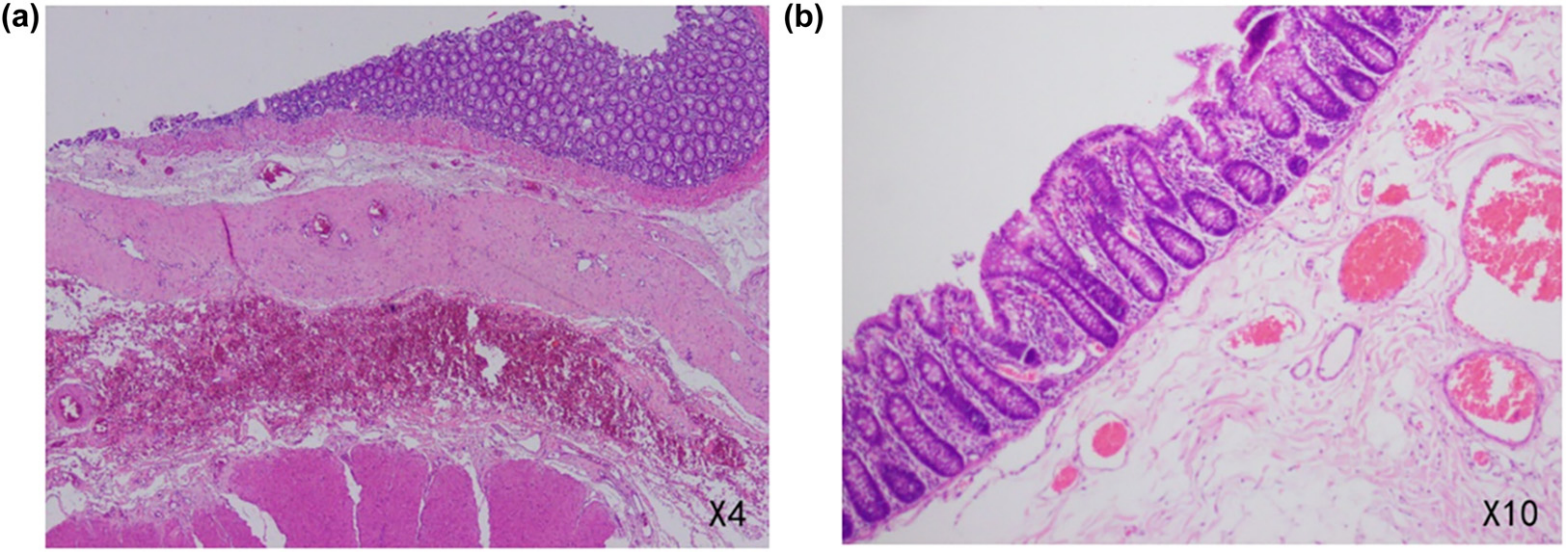
Microscopic examination of the transverse colonic duplication. (a) The layers of intestinal tubes are clear, and another myenteron can be seen on the other side of the serosa, suggesting that the two intestinal tubes share the same serosa and vessel. (b) Full-thickness vasodilation of the intestinal canal and reactive proliferation of lymph nodes around the intestine.

Microscopic examination of the dilated segment revealed chronic inflammatory changes in the mucosa on the surface, with an uneven thickness of the muscularis propria and irregular arrangement in some areas. The serosa was adhered to another intestinal segment, with an unclear structural delineation of the intestinal wall at the adhesion site. The entire layer of the intestinal wall showed dilation and hyperemia, with infiltration of a small number of chronic inflammatory cells around it and reactive hyperplasia of lymph nodes around the intestine. Finally, the diagnosis of transverse colonic duplication was confirmed (Figure 3).

Symptomatic treatment was administered postoperatively, and the patient recovered successfully. She was discharged after 10 days and followed up for 6 months without complications.

## 3 Discussion

Intestinal duplication is a rare congenital malformation characterized by the presence of a circular or tubular cavity organ on the mesenteric side, closely related to the adjacent intestinal blood supply [7]. It has been reported that approximately 0.01% of 10,000 newborns have gastrointestinal duplication [8,9]. The exact pathogenesis of intestinal duplication is not fully understood, and several hypotheses have been proposed. The first is the aberrant lumen recanalization theory: During early embryo recanalization, some vacuoles are not completely fused with the intestinal lumen, and there is a parallel interval with the digestive tract, which can then develop into adjacent intestinal tracts [10]. The second is the diverticular residue theory: There are many diverticula-like outer pockets in the digestive tract in the early embryo. Under normal circumstances, diverticula will disappear by gradual degeneration with the body’s growth. If some diverticula do not degenerate or have repeated malformations during the development process, they may develop into cystic intestinal duplication [11]. The third is the notochord developmental disability theory: The yolk sac protrudes during development, or the endoderm is pulled due to adhesion between the endoderm and ectoderm. When the endoderm develops into the intestinal canal, it can develop into various forms of digestive tract duplication [12]. Additionally, environmental factors, such as trauma and hypoxia, have also been implicated in the development of intestinal duplication. Due to trauma or hypoxia, ischemic necrosis occurs in the intestine, and the remaining intestinal fragments receive blood supply from nearby vessels, which can develop into intestinal duplication [11]. In our case, the patient had a history of a traffic accident 22 years ago, and trauma could not be ruled out as a possible cause of transverse colonic duplication.

Currently, intestinal duplication is classified into two main types: cystic and tubular. Cystic duplication is the most common type, accounting for approximately 80% of the cases [13]. The case reported in this article was an extraintestinal cystic duplication of the transverse colon. The cyst is oval and located on one side of the mesentery, communicating with the intestinal cavity, resulting in compression of the transverse colon and subsequent intestinal obstruction.

Intestinal duplication can occur at any location along the digestive tract, resulting in a wide range of clinical manifestations. These may include symptoms and signs associated with the specific location of the duplication, and in some cases, malignant changes may also occur. In a study by Inoue and Nakamura, colonic duplication is found to be relatively rare, accounting for 6.8% of all digestive tract duplications, but it has the highest proportion of cases diagnosed as malignant tumors, accounting for 67% [14]. Fortunately, no malignant cells were observed in the case of transverse colonic duplication reported in this work.

Diagnosing intestinal duplication is extremely challenging due to its complex anatomical structure and non-specific symptoms. Ultrasonography is one of the most commonly used methods for diagnosis. Typical intestinal duplication may result in double walls, the so-called “intestinal signals.” The inner layer is the hyperechoic mucosa, and the outer layer is the hypoechoic muscular layer. However, inflammation or other factors may cause blurring and difficulty distinguishing the layers [15,16]. CT examination has limited diagnostic value for intestinal duplication, but it can provide information on the relationship between the duplication and adjacent intestine, aiding in preoperative evaluation and reducing surgical risks. On CT, intestinal duplication usually appears as a fluid-filled mass with a thick wall and a slightly enhanced signal [17]. Recent studies have shown that endoscopic ultrasonography is more accurate for diagnosing intestinal duplication [18]. However, the transverse colon duplication is more difficult to diagnose. In a previous report, a case of transverse colonic duplication is initially misdiagnosed as a cystic pancreatic tumor [19]. Similar to our case, we considered other possible diagnoses, such as colonic diverticulum, megacolon, and congenital intestinal malformation, based on imaging findings, but an accurate judgment could not be made until the surgery. Due to the rarity and complex anatomical structure of intestinal duplication, it should be differentiated clinically from the following diseases: (1) Mesenteric cysts: Mesenteric cysts are mostly found in congenital malformations or traumas and are located between mesenteric lobules, with the ileum being the most common location. Compared to intestinal duplication, mesenteric cysts have a thin capsule wall, lack a muscular layer, have a complete capsule, and have no communication with the intestinal cavity. The clinical symptoms of mesenteric cysts typically encompass internal hemorrhage within the cyst, abdominal distension, abdominal pain, vomiting, and abdominal mass. A few patients suffer from anemia and intestinal obstruction. Ultrasonography and CT examination can be helpful for diagnosis [20,21]. (2) Meckel’s diverticulum: Meckel’s diverticulum, also known as congenital ileum end diverticulum, is a true diverticulum caused by incomplete degeneration of the vitelline duct and an unclosed intestine, mostly located in the ileum, approximately 100 cm from the ileocecal valve. Compared with intestinal duplication, Meckel’s diverticulum has an independent blood supply, while the intestinal duplication blood supply is most closely related to the surrounding intestine [3]. Some patients with Meckel’s diverticulum can be asymptomatic for life and are difficult to diagnose. Only 4–7% of the patients have been found with various complications, such as hematochezia, intestinal obstruction, and inflammation [22,23,24]. Isotope scanning with 99 mTc, which has a special affinity for the gastric mucosa and can be absorbed by it, can be used to diagnose Meckel’s diverticulum [25].

Currently, there are no specific guidelines for managing intestinal duplication. Based on relevant literature, surgery remains the preferred treatment [1,3,4,7,8,11,13]. Early surgical treatment is recommended for patients with intestinal obstruction and gastrointestinal bleeding. For patients with mild symptoms who cannot be completely diagnosed with intestinal duplication deformity, it is necessary to make an early diagnosis through a variety of examination methods and to choose a suitable time for surgery, considering the risk of tumor arising. Surgical methods include traditional open surgery and minimally invasive surgery, with the choice of technique depending on the location, size, type, and surrounding tissues of the duplication. During the operation, the intestinal blood supply should be protected.

In conclusion, colonic duplication is a rare occurrence, especially in adults. Diagnosing and treating colonic duplication are still significant clinical challenges, and surgical treatment is still the most important method. Other postoperative treatments should be further determined according to the patient’s specific conditions and postoperative pathology to relieve the patient’s clinical symptoms and improve their quality of life.
